# Evaluation of the efficacy of transcatheter arterial embolization combined with apatinib on rabbit VX2 liver tumors by intravoxel incoherent motion diffusion-weighted MR imaging

**DOI:** 10.3389/fonc.2022.951587

**Published:** 2022-09-13

**Authors:** Can Chen, Xiao Liu, Lingling Deng, Yunjie Liao, Sheng Liu, Pengzhi Hu, Qi Liang

**Affiliations:** ^1^ Department of Radiology, The Third Xiangya Hospital, Central South University, Changsha, China; ^2^ Teaching and Research Section of Imaging and Nuclear Medicine, The Third Xiangya Hospital, Central South University, Changsha, China

**Keywords:** liver neoplasms, transcatheter arterial embolization, apatinib, angiogenesis, intravoxel incoherent motion

## Abstract

**Background and purpose:**

It is crucial to evaluate the efficacy, recurrence, and metastasis of liver tumors after clinical treatment. This study aimed to investigate the value of Introvoxel Incoherent Motion (IVIM) imaging in the evaluation of rabbit VX2 liver tumors treated with Transcatheter Arterial Embolization (TAE) combined with apatinib.

**Methods:**

Twenty rabbit VX2 liver tumor models were established and randomly divided into either the experimental group (n=15) or the control group (n=5). The experimental group was treated with TAE combined with oral apatinib after successful tumor inoculation, while no treatment was administered following inoculation in the control group. IVIM sequence scan was performed in the experimental group before treatment, at 7 and 14 days after treatment. All rabbits were sacrificed after the last scan of the experimental group. Marginal tissues from the tumors of both groups were excised for immunohistochemical analysis to observe and compare the expression of microvessel density (MVD). The alterations of IVIM-related parameters of tumor tissues in the experimental group, including Apparent Diffusion Coefficient (ADC), True Diffusion Coefficient (D), Pseudodiffusion Coefficient (D*), and Perfusion Fraction (*f*) were compared at different periods, and the correlation between these parameters and MVD was analyzed.

**Results:**

After treatment, ADC and D values significantly increased, whereas D* and *f* values both decreased, with statistically significant differences.(*P*<0.05). The average tumor MVD of the experimental group after TAE combined with apatinib ((33.750 ± 6.743) bars/high power field (HPF)) was significantly lower than that in the control group ((64.200 ± 10.164) bars/HPF)). Moreover, D and *f* were positively correlated with tumor MVD in the experimental group (*r*=0.741 for D and *r*=0.668 for *f*, *P*<0.05). However, there was no significant correlation between ADC and D* values of the experimental group and tumor MVD (*r*=0.252 for ADC and *r*=0.198 for D*, *P*>0.05).

**Conclusion:**

IVIM imaging can be employed to evaluate the efficacy of TAE combined with apatinib in rabbit VX2 liver tumors. Alterations in D and *f* values were closely related to the MVD of liver tumor tissues.

## Introduction

Primary hepatocellular carcinoma(HCC) is the sixth most commonly diagnosed cancer and the third-leading cause of cancer-related deaths worldwide. The incidence rate in China is as high as 10-20 per 100,000 people, accounting for about 45% of the global mortality rate (1, 2). Transcatheter arterial chemoembolization (TACE) combined with oral sorafenib is the standard treatment for patients with advanced liver cancers that cannot be surgically resected (3). Apatinib is a novel small-molecule anti-angiogenic targeted drug independently developed in China, which can highly selectively inhibit VEGFR-2 receptors with 10 times the binding affinity of sorafenib. It can also inhibit multiple ATP binding sites, thus having the advantage of efficiently reversing drug resistance. In recent years, it has been widely used in the anti-angiogenesis of tumors due to its benefits, such as low cost, good therapeutic effect, and low drug resistance (4, 5).

Most patients with liver cancer need multiple sessions of TAE, therefore, accurate and repeated postoperative evaluation of the tumor focus is a key factor for the timely detection of survival and recurrence of cancer (6, 7). Intravoxel incoherent motion (IVIM) diffusion-weighted imaging (DWI) can distinguish the diffusion of water molecules in tissue lesion areas and microperfusion of the local capillary network, and estimate the Apparent Diffusion Coefficient (ADC), True Diffusion Coefficient (D), Pseudodiffusion Coefficient (D*) and Perfusion Fraction (*f*), which can be used to quantitatively distinguish surviving and necrotic tumor tissues at an early stage by multiple parameters (8, 9). Herein, the expression of microvessel density (MVD) of tumor tissues in treated and non-treated groups were compared, and the changes of IVIM imaging-related parameters before and after TAE combined with apatinib treatment of VX2 liver tumors in rabbits were analyzed to explore the relationship between IVIM imaging-related parameters and the histopathological MVD, aiming to assess the value of IVIM imaging in evaluating the therapeutic effect of TAE combined with apatinib.

## Methods

### Materials

Twenty healthy and clean New Zealand white male rabbits, weighing (3.00 ± 0.25) kg and aged 3 to 5 months, were provided by The Animal Experiment Center of The Third Xiangya Hospital of Central South University. Other materials included Apatinib mesylate tablets (0.25g/tablet,Jiangsu Hengrui Medicine Co., Ltd.,Liangyungang city,China), Iohexol Injection (300 mg I/ml, GE Healthcare Shanghai Co.,Ltd.,Shanghai,China), a complete dyeing system of CD34 antibody, 4-F Cobra catheter, 3% Pentobarbital Sodium and related interventional operation instruments, etc.

### Rabbit VX2 liver tumors modeling

Tissues at the edge of the tumor from tumor-bearing rabbits were sliced into 1-2 mm^3^ sections for implant. The rabbit VX2 liver tumor model was established by traditional laparotomy (10). A 3 cm incision was made along the linea alba to expose the left lobe of the liver under strict aseptic conditions. A small incision of about 2 mm was made on the left lobe of the liver with ophthalmic scissors, and the tumor subsequently was implanted. Afterward, the incision was closed with a cotton glue sponge, and the abdominal wall was routinely sutured. After surgery, 200,000 units of penicillin were injected intramuscularly for 3 days to prevent infection.

### Grouping and TAE process

Rabbits were divided into either the experimental group (n=15) or the control group (n=5) using the random number method. In the experimental group, TAE was performed 2 weeks after inoculation: super-selective insertion into the tumor target artery, 0.4 mg microspheres were injected to embolize the blood vessel, and then transcatheter angiography was performed again to determine tumor embolization. After surgery, penicillin was intramuscularly injected for 3 days to prevent infection. The apatinib suspension was prepared with normal saline. Within 24 hours after TAE, the experimental rabbits were orally fed apatinib (50 mg/kg/d) for the first time with a self-made syringe combined with a soft hose, then once a day for 2 consecutive weeks. For the control group, no treatment was administered following inoculation.

### IVIM scanning and image post-processing

Axial T1-weighted, T2-weighted, and IVIM sequence scans were performed by 3.0T MR (Ingenia, Philips Healthcare, Netherlands) before TAE and on days 7 and 14 after TAE. The rabbits were fasted for 12 hours on the examination day, fixed supinely on the examination bed under general anesthesia, and scanned with a human knee coil. A total of 12 b values (0, 10, 20, 30, 40, 50, 75, 100, 150, 300, 500, and 800) were selected for IVIM-DWI scanning. Scan parameters: TE=55.8 ms, TR=1099 ms; FOV: AP/PL/FH: 300/179/171 mm; Matrix M×P: 100×58; Thickness/spacing: 3.0/0 mm; ACQ Voxel MPS (mm): 3.00/3.01/6.00; NSA = 1.

The Philips professional post-processing software was utilized for the post-processing of images. The collected DICOM image data were imported to generate IVIM parameter images: ADC, D, D*, and *f.* Afterward, the Region of Interest (ROI) was manually constructed according to T2WI images and the obvious diffusion restricted areas on DWI images. ROI was drawn as far as possible on the layer with the largest and most apparent solid component in the lesion. Data from the same lesion were measured three times, and the average value was taken. Finally, the data were exported, and the corresponding ADC, D, D*, and *f* pseudo-color images were constructed.

### Pathology and immunohistochemistry

**Figure 1:**
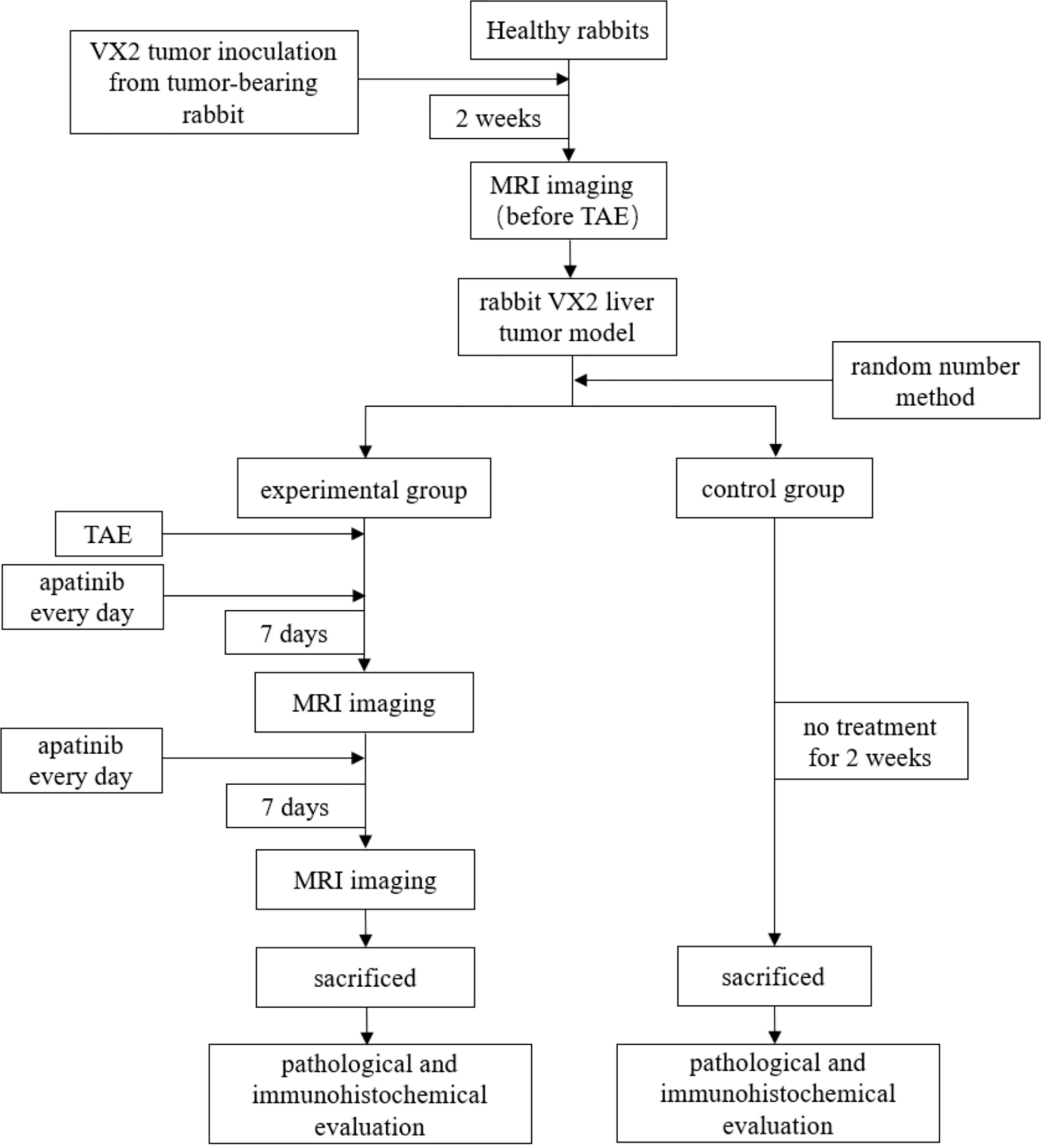
The workflow of this study.

Rabbits in the two groups were sacrificed after the last scan of the experimental group. The whole liver was isolated, and the location of the target tumor was determined. Then, the tumor and its surrounding tissues were dissected and dispatched to the pathology department for paraffin embedding and sectioning. As far as possible, sections of the same layer as the scanning layer were selected for HE staining and CD34 immunohistochemical staining, and MVD expression was observed and counted by pathologists. The workflow of this study is listed in **Figure 1**.

Calculation of MVD: the brownish-yellow color in the cytoplasm of CD34 immunohistochemical staining was considered a positive expression, representing vascular cells. The WEIDNER method was used for counting (11): Five vascular regions with the highest density of staining were randomly selected under low power (100 times) light microscope, and the target regions were subsequently counted under high power (400 times) light microscope. The average of the 5 target regions selected was recorded as the MVD value [bars/high power field(HPF)] of the case.

### Statistical analysis

Statistical analyses were performed with statistical software (SPSS, version 22.0). All numerical variables were expressed as (x ± s). In contrast, the analysis of variance (ANOVA) was used for multi-group comparison, and the t-test was used for comparison of each two group of the same parameters. Pearson correlation analysis was conducted on the correlation of IVIM-related quantitative parameters ADC, D, D* and *f* with MVD, and *P*<0.05 was considered statistically significant.

## Results

### Tumor inoculation and TAE results

A total of 20 rabbits were inoculated with the tumors. During the two-week observation period, tumor-bearing rabbits grew normally without infection or other complications. Two weeks after inoculation, 20 cases were confirmed to be successfully inoculated using the MR scan, hence a success rate of 100%. The mean tumor diameter measured on MR images was approximately (1.35 ± 0.21) cm.

**Figure 2:**
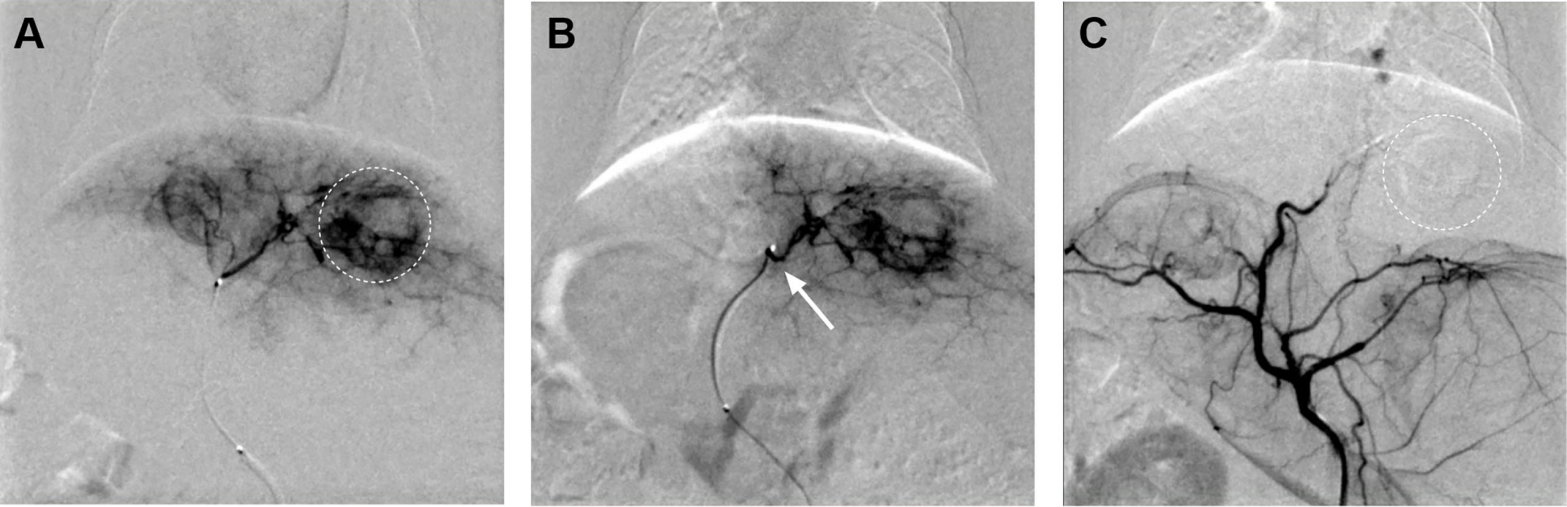
**(A)** Hepatic artery angiography exhibited abundant tumor blood supply (dotted circle). **(B)** The catheter tip was super-selected to the tumor supplying artery of the left hepatic artery (arrow). **(C)** The tumor-supplying artery disappeared after TAE.

In the experimental group, 15 tumor-bearing rabbits were treated with TAE embolization by modified femoral artery intubation, and the success rate was 100%. DSA images displayed obvious nodules or clumps, with uneven staining at the arterial stage, presenting the bulbous sign. After embolization, the supplying artery of the tumor disappeared, and the mass did not develop (**Figure 2**). However, one rabbit died three days after surgery. It was observed that the dead rabbit had a large area of gray infarct in the liver and intestinal obstruction in the abdominal cavity.

### The tumor changes at 7d and 14d after TAE

**Figure 3:**
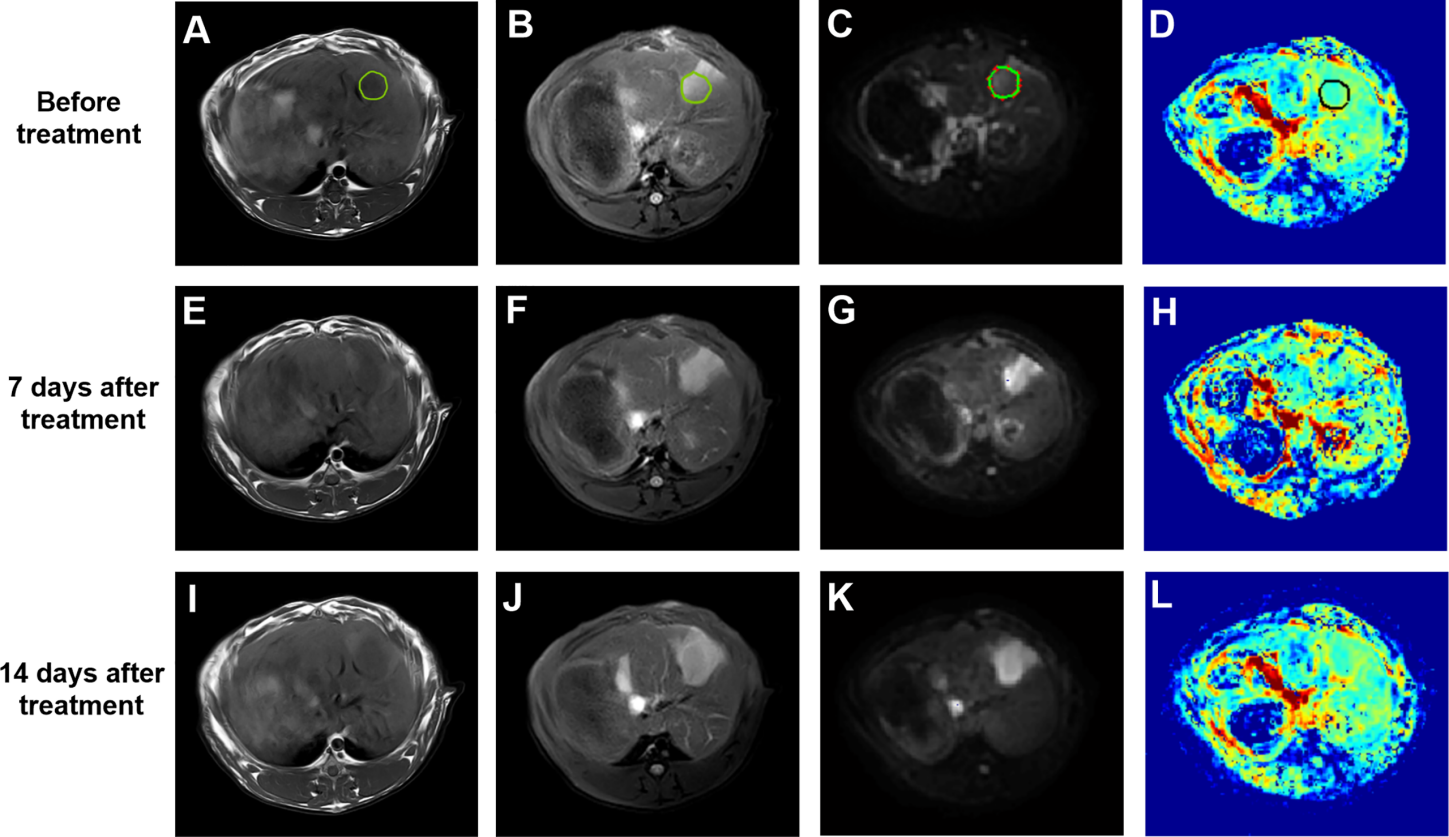
**(A–D)** T1WI, T2WI, DWI (b=500 s/mm^2^) and pseudo-color image of D before treatment, the green or black circle is the area where the tumor was planted. **(E–H)** T1WI, T2WI, DWI (b=500 s/mm^2^) and pseudo-color image of D 7 days after treatment. **(I–L)** T1WI, T2WI, DWI (b=500 s/mm^2^) and pseudo-color image of D 14 days after treatment. DWI, diffusion-weighted imaging; D, true diffusion coefficient.

VX2 liver tumors showed a slightly low signal on T1WI, slightly high signal on T2WI, significantly high signal on DWI, decreased signal on the ADC image, and unclear boundaries with surrounding normal liver tissues. With TAE combined with apatinib treatment, the tumor center was gradually necrotic, and the D value of reacting water diffusion increased compared with that before treatment. The mean tumor diameter measured on MR images was approximately (1.48 ± 0.38) cm on day 7 after treatment. On day 14, diffuse and limited tissues were still visualized at the tumor margins on DWI images, suggesting limited residual tumor tissues (**Figure 3**).

### IVIM-related parameters of tumor margins at 7 and 14d after TAE

**Figure 4:**
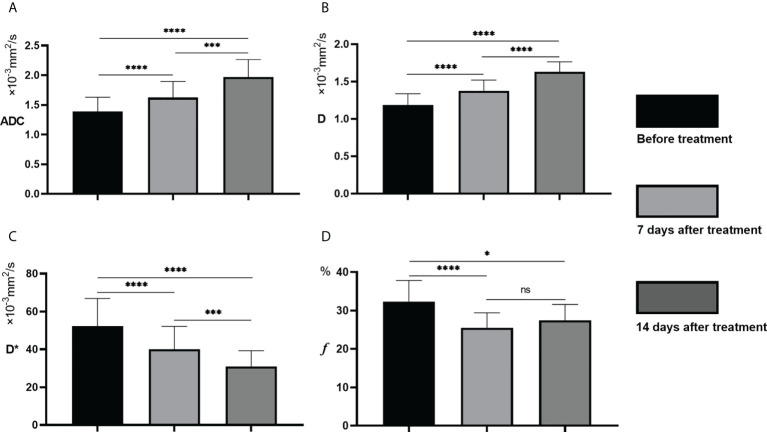
Change trend of ADC**(A)**, D**(B)**, D*(C) and *f* **(D)** before and after treatment. ns represents *P* >0.05,*P < 0.05, ***P < 0.001, ****P < 0.0001.

**Figure 5:**
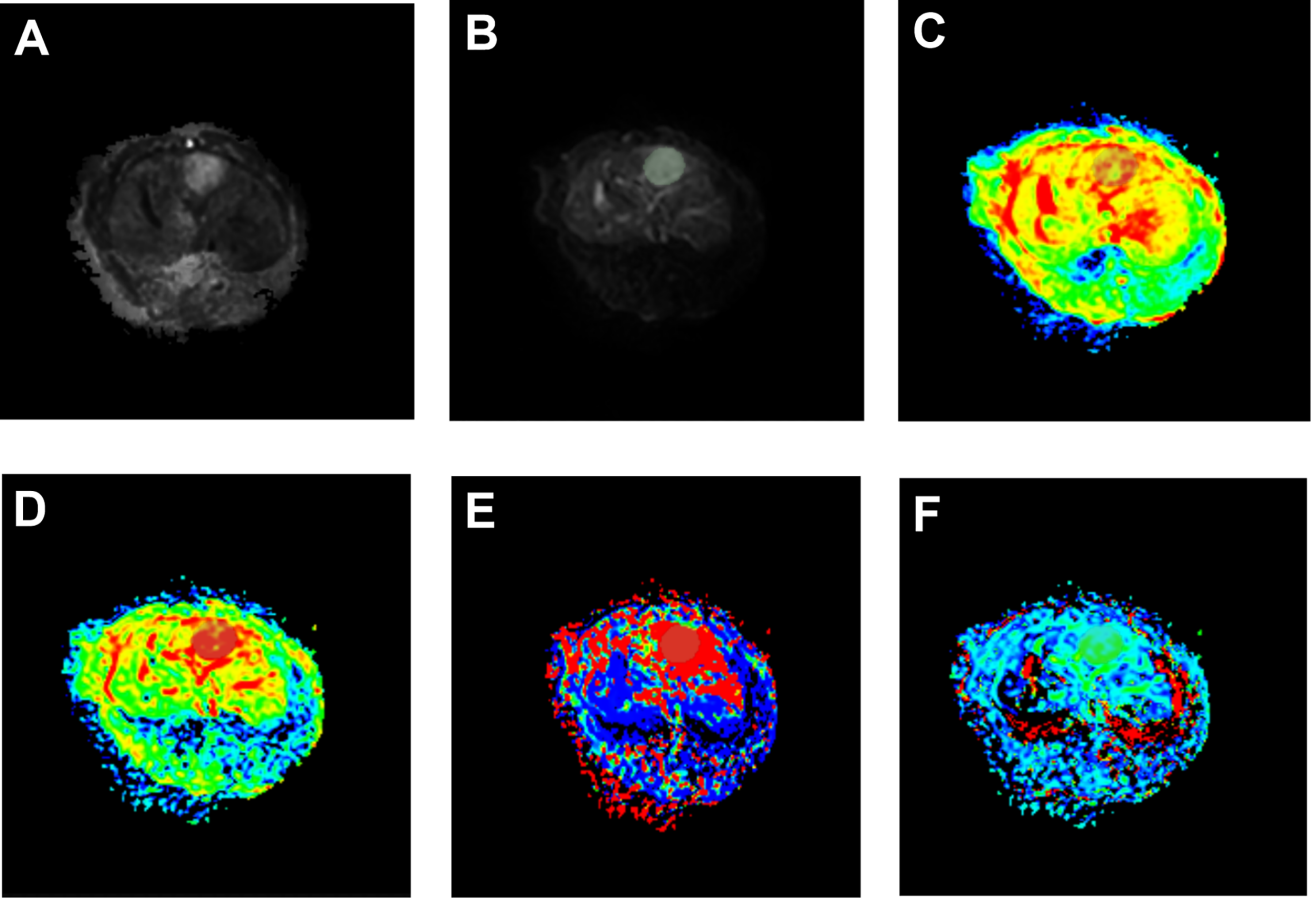
Pseudo-color images of IVIM-related parameters 7 days after TAE combined with apatinib treatment. The tumor showed a significantly high signal on raw DWI images **(A)**, post-processed DWI images **(B)**. In pseudo-color images of ADC **(C)**, pseudo-color images of D **(D)**, pseudo-color images of D* **(E)**, and pseudo-color images of *f* **(F)**, the grey shaded areas were limited diffusion areas and ROI areas of the tumor.

The IVIM-related parameters of tumor margins in the experimental group before treatment and on the 7th and 14th day after treatment are presented (**Table 1**). On the whole, ADC and D values increased, while D* and *f* values decreased after treatment, and the differences were statistically significant (*P<0.05*). In further pairwise comparison of the same parameter before and after treatment, there was only no statistical difference between *f* values on the 7th and 14th day after treatment (**Figure 4**). Pseudo-color images of IVIM-related parameters7days after TAE combined with apatinib treatment were showed in **Figure 5**.

**Table 1:** Comparison of IVIM-related parameters in the experimental group before and 7 and 14 days after TAE combined with apatinib treatment (x ± s).

Time	Numberof tumors	ADC	D	D*	*f*
	(n)	(×10^−3^ mm^2^/s)	(×10^−3^mm^2^/s)	(×10^−3^ mm^2^/s)	(%)
before treatment	14	1.387 ± 0.243	1.188 ± 0.150	52.128 ± 14.792	32.366 ± 5.452
7 days after treatment	14	1.623 ± 0.273	1.377 ± 0.144	40.057 ± 12.072	25.543 ± 3.875
14 days after treatment	14	1.967 ± 0.295	1.629 ± 0.134	31.050 ± 8.206	27.449 ± 4.149
*P*		<0.05	<0.05	<0.05	<0.05

ADC, apparent diffusion coefficient; D, true diffusion coefficient; D*, pseudodiffusion coefficient; f, perfusion fraction.

### Histopathological results

**Figure 6:**
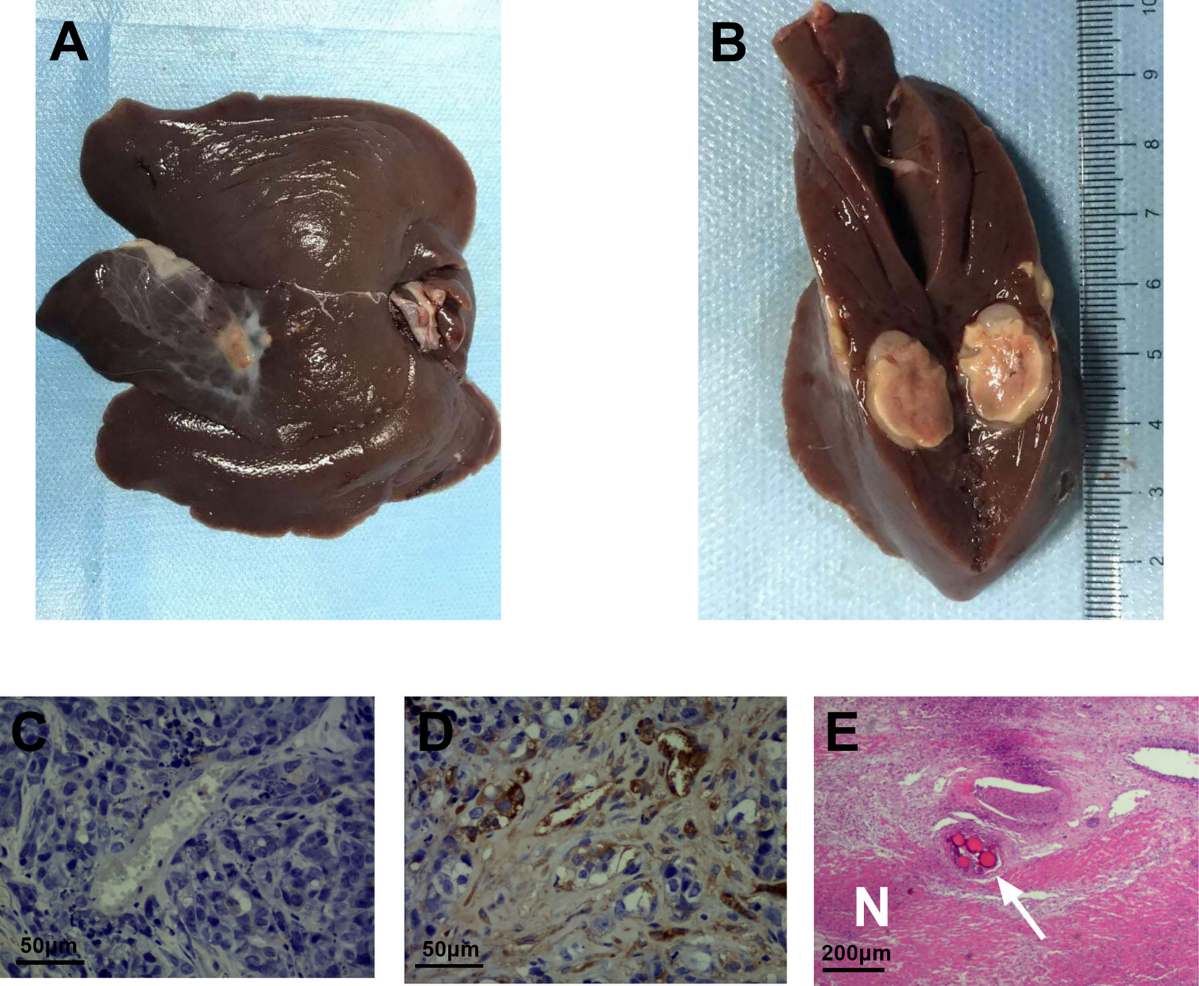
CD34 immunohistochemical staining and HE staining of the experimental and control groups. **(A)** pathological tissues from the experimental group. **(B)** coagulative necrosis was present in the center of the tumor, a fish-flesh appearance at the edge of the tumor. **(C)** CD34 immunohistochemical staining of the experimental group showed a few scattered brownish-yellow cells(400 times). **(D)** CD34 immunohistochemical staining of the control group still exhibited a large number of brownish-yellow cells(400 times). **(E)** the necrotic area (N) and embolization of microspheres in blood vessels (arrow) can be visualized by HE staining of the experimental group(100 times).

The pathological tissues from the experimental group showed that coagulative necrosis was present in the center of the tumor, a fish-flesh appearance at the edge of the tumor (Figures 6A, B). The positive expression representing vascular cells of the experimental group **(** **Figure 6C** **)** were significantly reduced compared with the control group **(** **Figure 6D** **)**. HE staining revealed extensive coagulation necrosis in the center of the tumor and embolic microspherules clustered in the vascular area **(** **Figure 6E** **).** The mean MVD of the experimental group was (33.750 ± 6.743) bars/HPF, while that of the control group was (64.200 ± 10.164) bars/HPF, indicating that the expression of MVD was significantly decreased after TAE combined with apatinib compared with that of the control group (*t*=7.604, *P* < 0.01).

### Correlation analysis of IVIM-related parameters and MVD

**Figure 7:**
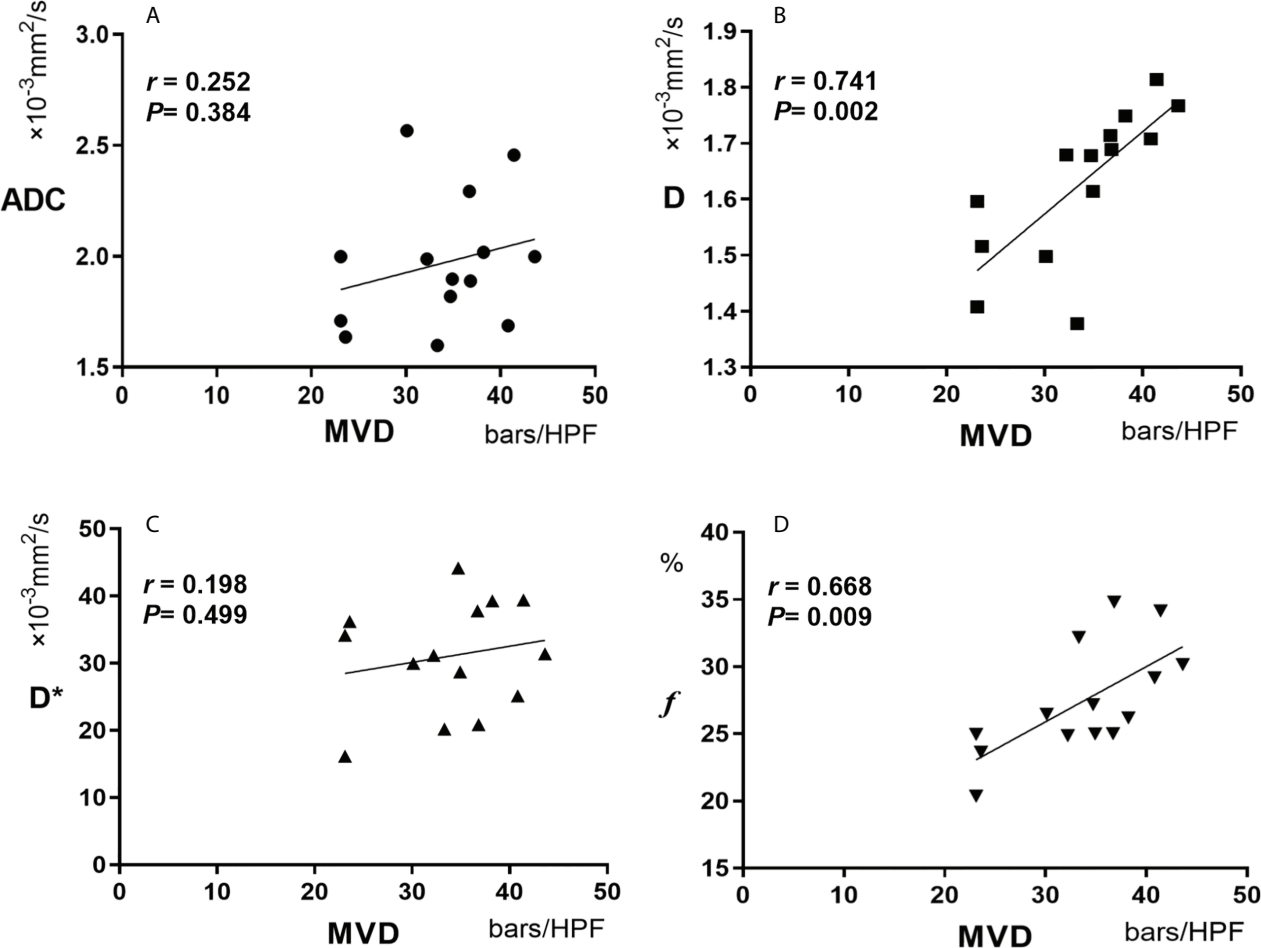
Pearson correlation between MVD and ADC **(A)**, D**(B)**, D*(C) and *f* **(D)**. MVD, microvessel density.

Spearman correlation analyses revealed that IVIM-related parameters D and *f* were positively correlated with tumor MVD in the experimental group (*r*=0.741 for D and *r*=0.668 for *f*, *P<0.05*). However, ADC and D* were not significantly correlated with tumor MVD (*r*=0.252 for ADC and *r*=0.198 for D*, *P>0.05*) **(** **Table 2** and **Figure 7** **)**.

**Table 2:** The correlation between IVIM-related parameters and MVD in the experimental group after 14 days of TAE combined with apatinib treatment.

IVIM relatedparameters	MVD(bars/HPF)	
	*r*	*P*
ADC	0.252	0.384
D	0.741	0.002
D*	0.198	0.499
*f*	0.668	0.009

## Discussion

Since the introduction of diffusion-weighted imaging (DWI), tremendous success has been achieved in the quantitative study of the diffusion coefficient ADC, especially in differentiating benign and malignant tumors and pathological grades, such as hepatocellular carcinoma and breast cancer (12–14). However, previous studies have shown that the ADC values obtained by a simple exponential relationship on DWI sequences could not distinguish water molecular diffusion from blood perfusion (15, 16). In this study, the D value of VX2 liver tumors was always lower than the ADC value, which signaled that the ADC value included the component of blood perfusion, which was consistent with the results reported by Yoon JH et al (17). On the 7th day after embolization in the experimental group, ADC and D values that can reflect the diffusion of water molecules in tissues increased significantly, while the D* and *f* values that can reflect the perfusion of blood decreased significantly. This is because TAE can significantly reduce the blood perfusion of tumor tissues by directly blocking the main supplying vessels of the tumor. Besides, the antivascular effect of apatinib reduces the generation of new blood vessels and affects the perfusion of tumor tissues, thus achieving a dual effect of inhibiting tumor growth (18, 19). This result is consistent with several studies that determined that postoperative perfusion volume of lesions in HCC patients treated with TAE decreased significantly compared with that before surgery (20, 21).

In recent years, IVIM imaging technology has been widely studied in the differential diagnosis of liver tumors, alternative pathological grading and degree of cirrhosis (22, 23). IVIM imaging is a non-invasive functional imaging method to evaluate tumor angiogenic perfusion and diffusion of water molecules in tumors. Therefore, IVIM imaging technology can theoretically be used as an effective means to evaluate the efficacy of TAE combined with anti-angiogenic therapy for VX2 liver tumor. IVIM-related parameters D* and *f* both reflect the blood perfusion of the capillary network. Consequently, the changing trend of the two should be theoretically consistent. However, the results of this study found that the *f* value was no longer decreasing on the 14th day compared with the 7th day, while D* continued to decline, showing inconsistent changes between the two. Previous literature showed that *f* mainly reflects capillary capacity, while D* is mainly determined by capillary blood flow velocity and length. Therefore, *f* and D* changes can be inconsistent given that they reflect different aspects of blood perfusion (24).It was also clarified that the formation of HCC is mainly characterized by arterialization changes in blood supply, that is, increased hepatic artery blood supply and increased new vessels, which may affect the consistency of D* and *f (* 25). However, the main limitations of this study were that the time window was short and the sample size was relatively small. Whether the variation in *f* is consistent with the change in D* remains to be further verified.

The function and state of microvessels in tumor tissues are directly related to tumor growth, invasion, metastasis, and prognosis (26). Therefore, it is necessary to clarify the relationship between perfusion changes and tumor angiogenesis on imaging. Currently, MVD remains the histological gold standard for evaluating tumor angiogenesis. In this study, the average MVD of the experimental group was (33.750 ± 6.743) bars/HPF, while that of the control group was (64.200 ± 10.164) bars/HPF, indicating that the expression of MVD in the experimental group was significantly lower than that in the control group. TAE combined with apatinib can inhibit tumor formation by directly blocking the main supplying vessels of the tumor and inhibiting tumor angiogenesis, which is consistent with the results of other studies that inhibition of vascular endothelial growth factor (VEGF) expression can promote tumor cell apoptosis and cell necrosis (27).

This study found that the IVIM-related parameters D and *f* were positively correlated with tumor MVD (*r*=0.741 for D and *r*=0.668 for *f*, *P*<0.05). However, ADC and D* had no significant correlation with tumor MVD (*r*=0.252 for ADC and *r*=0.198 for D*, *P*>0.05). As mentioned above, ADC includes water molecular diffusion of tissues and vascular microcirculation perfusion of the capillary network, so ADC may not be the most accurate biomarker for the pathophysiological behavior of tissues. In addition, D represents the diffusion of pure water molecules in the voxel. There should theoretically be no correlation between D and MVD. Still, our results found that D was correlated with MVD, which may be related to the following reasons: Firstly, VX2 tumor is rich in immature neovascularization with high permeability and extensive abnormal pathways; Secondly, the region of MVD calculation was selected in the most apparent area of angiogenesis. Therefore, due to the unique characteristics of neovascularization in liver tumor cells, D, representing the diffusion of water molecules, showed a certain correlation with MVD. ADC is generally considered a combination of true molecular diffusion and microcirculation perfusion effect and therefore did not correlate with MVD.

D* is a parameter of microcirculation perfusion, and both this study and previous ones have established that the D* value was significantly higher than the D value (28). Herein, we also found no significant correlation between D* and MVD(*r*= 0.198). Therefore, we postulate that D* is not a valuable parameter to reflect the microcirculation perfusion of tumor tissues. However, this result is contrary to the results reported by Gulbay M et al. that D * is statistically correlated with MVD (*r*= 0.415) (29). The reasons may be related to the following factors: Firstly, D* has poor repeatability; Secondly, the liver is a double blood supply organ with complex microcirculation perfusion characteristics; Thirdly, a large number of immature neovascularization have high permeability and extensive abnormal pathways in VX2 liver tumors. Therefore, the role of D* in the evaluation of microcirculation perfusion needs to be further studied and validated. *f* is the perfusion fraction of the microvascular network, representing the volume ratio of perfusion effect caused by microcirculation in the voxel to the total diffusion effect. In this study, it was noted that *f* was correlated with MVD (*r*=0.668, *P*=0.009), which is consistent with the conclusions of several studies (30, 31). It is documented that *f* is a surrogate indicator of blood volume, and the vascular distribution of tumors affects the value of *f* (32). However, from the results of this study, *f* can be used to quantitatively evaluate the angiogenesis of tumor tissues, and thus may be used to evaluate the early efficacy of TAE combined with targeted drugs in the treatment of liver tumors.

There are certain limitations to this study. First, the sample size of experimental animals was relatively small. Secondly, histopathological analyses were performed manually, which may be biased. Thirdly, our study only focused on the tumor angiogenesis without evaluating the changes of tumor size after treatment, which may also be an important indicator reflecting the efficacy of treatment. Finally, this study was limited to the animal model, so it cannot be considered to have identical effects as human liver experiments.

## Conclusion

To conclude, our study showed that IVIM imaging is a non-invasive and effective examination for quantitative analyses of the microscopic pathophysiological status of tumors. IVIM-related parameters D and *f* may be used to evaluate the efficacy of TAE combined with apatinib in the treatment of rabbit VX2 liver tumors so as to provide vital information for the later clinical diagnosis and treatment of liver tumors.

## Data availability statement

The original contributions presented in the study are included in the article/supplementary material. Further inquiries can be directed to the corresponding author.

## Ethics statement

The animal study was reviewed and approved by the medical ethics committee of the Third Xiangya Hospital of Central South University.

## Author contributions

All authors contributed to study design. CC, XL, and LD contributed to data collection and analysis. SL, PH, and QL contributed to operation guide. CC, XL, and LD contributed to the statistics. CC and QL contributed to manuscript writing and revision. All authors contributed to the article and approved the submitted version.

## Conflict of interest

The authors declare that the research was conducted in the absence of any commercial or financial relationships that could be construed as a potential conflict of interest.

## Publisher’s note

All claims expressed in this article are solely those of the authors and do not necessarily represent those of their affiliated organizations, or those of the publisher, the editors and the reviewers. Any product that may be evaluated in this article, or claim that may be made by its manufacturer, is not guaranteed or endorsed by the publisher.
